# Successful treatment of an 82‐year‐old COVID‐19 delta variant‐infected patient with haemorrhagic stroke and active prostate cancer brain metastasis

**DOI:** 10.1002/ctd2.20

**Published:** 2022-01-12

**Authors:** Buhai Wang, Jiangquan Yu, Yichun Zeng, Xian Zhang, Chongxu Han, Qili Lu, Xiaolin Wang, Yichen Liang, Juan J. Gu, Yusheng Shu

**Affiliations:** ^1^ Department of Thoracic Surgery Northern Jiangsu People's Hospital Yangzhou 255000 China; ^2^ Department of Oncology Northern Jiangsu People's Hospital Yangzhou 255000 China; ^3^ Cancer Institute affiliated to Northern Jiangsu People's Hospital Yangzhou 255000 China; ^4^ Medical College Yangzhou University Yangzhou 255000 China; ^5^ Intensive Care Unit Northern Jiangsu People's Hospital Yangzhou 255000 China; ^6^ Department of Clinical Laboratory Northern Jiangsu People's Hospital Yangzhou 255000 China; ^7^ Medical Service Division Yangzhou Third People's Hospital Yangzhou 255000 China

Dear Editor,

The pandemic coronavirus disease 2019 (COVID‐19) infection caused by severe acute respiratory syndrome coronavirus 2 (SARS‐CoV‐2), especially B.1.617.2 (delta variant), has catastrophic impact over the world started from December 2019.^1^, ^2^, ^3^ Cancer patients are immunosuppressed and have high severity and mortality rate of COVID‐19 infection^4^; therefore, we present a successful case of an 82‐year‐old man who was infected with COVID‐19 delta variant and severe concomitant complications.

An 82‐year‐old male presented to the emergency department who had fallen at home. In the preceding 3 days, the patient had progressive confusion, obtundation, slurred speech and difficulty with word finding. The past medical history includes stage IV prostate cancer with brain metastasis for 6 years, pulmonary emphysema, hypertension and coronary atherosclerotic heart disease with coronary stenting for 1 month. The patient was under active therapy for late‐stage brain metastasis of prostate cancer. He daily took abiraterone 1000 mg, rosuvastatin 5 mg, bayaspirin enteric‐coated 100 mg, clopidogrel 75 mg and felodipine 10 mg. He took betaloc 25 mh twice a day.

The patient was real time‐PCR (RT‐PCR) confirmed positive for the COIVD‐19 delta variant. He had a history of public gathering and a potential contacting people tested positive for COVID‐19. Physical examination was unremarkable.

His vitals were as follows: heart rate 88 beats/min, blood pressure 105/79 mm Hg, respiration rate 19 breaths/min and oxygen saturation 95%. Initial head computed tomography (CT) showed that several solid masses were seen in temporal horn of the right lateral ventricle, largest as 32 mm × 25 mm with calcified deposit. Intracerebral haematoma within the right lateral ventricle was seen with surrounding edema. Extra‐axial fluid collection without midline shift was also observed. The size of solid masses was consistent with previous head CT scan with diagnosis of prostate cancer brain metastasis. No new lesions and enlargement of the tumour was found. The chest CT scan showed multiple, bilateral ground‐glass opacities in the lower lobes consistent with COVID‐19 viral pneumonia. Pulmonary emphysema can also be seen on the chest CT (Figure 1). A mild pericardial effusion and a pleural effusion were observed on chest CT. The laboratory is summarized in Table 1. Chemistry panel showed hypokalaemia (2.62 mmol/L), high C‐reaction protein (93.6 mg/L) and high creatine kinase (2026 U/L).

**FIGURE 1:**
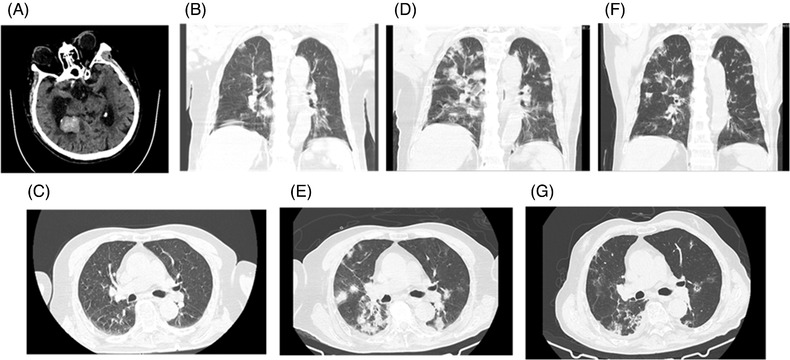
Head computed tomography and two chest CT studies in the coronavirus disease 2019 (COVID‐19) patients

**TABLE 1:** Summary of laboratory data (9 August, day 4)

Test	Result	Normal range
White blood cells	8.11	3.5–9.5 10^∧^9/L
Haemoglobin	133	130–175 g/L
Neutrophil	74.3	40–75%
Lymphocyte	17.8	20%–50%
Monocyte	7.8	3%–10%
Basophil count	0.01	0–0.06 10^∧^9/L
Eosinophil count	0.00	0.02–0.52 10^∧^9/L
Glucose	6.77	3.9–6.1 mmol/L
Sodium	137	137–147 mmol/L
Potassium	2.62	3.5–5.3 mmol/L
Chloride	93	137–147 mmol/L
Carbon Dioxide	29.3	22–29 mmol/L
Calcium	2	2.15–2.5 mmol/L
C‐response protein	93.6	<5.00 mg/L
Creatine kinase	2026	50–310 U/L
Alkaline Phosphatase	78	45–125 U/L
Total proteins	60.5	65–85 g/L
Albumin	37.0	40–55 g/L
Urea	9.3	3.6–9.5 mmol/L
Creatinine	108	44–133 umol/L
Total bilirubin	11.7	≤24 umol/L
Direct bilirubin	7.1	<5.0 mmol/L
Indirect bilirubin	4.6	0–20 mmol/L
Aspartate Aminotransferase (AST)	96.5	10–60 U/L
Alanine Aminotransferase (ALT)	30.5	9–50 U/L
Lactate Dehydrogenase (LDH)	533	0–25 U/L

He was hospitalized in the Intensive Care Unit (ICU) ward for COVID‐19 treatment and intracranial haemorrhage, following protocol from infectious disease for COVID‐19 positive patient. He was started on treatment with neutralizing anti‐COVID 19 antibody 2000 mg once on day 2, and supplementary oxygen was 4 L/min. He was on potassium supplement for correcting hypokalaemia. Osmotic mannitol was administered to reduce swelling of the brain. On day 3, the patient had small amount of black tarry fluid from the nasogastric tube and proton pump inhibitor omeprazole 40 mg daily was prescribed to the treatment of a stress ulcer. There was no black tarry fluid from nasogastric tube found from day 5.

The patient's neurological condition has been improved from day 5. From day 9, COVID 19 testing was consecutively negative for 3 days. On day 16, a repeat chest CT scan showed a significant resolution of the bilateral pulmonary opacities (Figure 1). The patient was then discharged from ICU and transferred to an in‐patient ward for further supporting treatment.

According to the studies on COVID‐19 alpha strain, people with certain conditions can get severe illness, including older adults cancer, chronic lung disease, neurological conditions, diabetes, coronary artery disease, HIV infection, liver disease, overweight, liver disease and smoking.^1^ Our patient had various high‐risk factors: elderly patient; concomitant late‐stage active prostate cancer; long‐term of anti‐cancer treatment; neurological condition of brain metastasis from prostate cancer; pre‐existing underline disease of the lung and heart; 1‐month post‐surgical condition of coronary stenting and intracerebral haemorrhage. All these risk factors indicated severe disease and higher mortality rate in patient with COVID‐19 delta variant infection.

Intracerebral haemorrhagic stroke is a common and lethal complication of COVID‐19 infection with reported incidence of 7.2% in a UK surveillance study.^5^ Angiotensin‐converting enzyme 2, a major target in SARS‐CoV‐2 infections, is highly expressed on vascular endothelial cells and the heart. COVID‐19 virus can attack the vascular system and cause bleeding.^6^ Neurologic complications were also commonly seen in patients with COVID‐19.^7^ Critical patients seem to have higher incidents of neurological involvement than patients who have mild disease.^8^ Since pre‐existing brain metastasis, our patient showed no new lesions. Haemorrhage on the head CT scan made COVID‐19 the most likely cause of neurologic dysfunction.

Patient neurological condition was improved from day 5. On the day 9, COVID 19 RT‐PCR was converted into negative. There were not many reported successful cases of the patient who was infected with COVID‐19 delta variant with various risk factors. Management is challenging because experience is limited from a few cases.

The severe complication of COVID‐19 disease in the patient was well controlled by early initiation of non‐competitive SARS‐CoV‐2 neutralizing antibody cocktail against receptor‐binding domain domain of SARS‐CoV‐2. This treatment can block entry of the virus, decrease the viral load, and therefore minimize the chance of causing the disease severity and improving recovery.^9^ Our patient was given a single infusion of neutralizing antibody cocktail BRII‐196 and BRII‐198 2000 mg on the second day after admitted, which was fall in the efficient timeline for the neutralizing antibody to reduce the viral loading. We believed this is one of the key factors which successfully rescued the patient from severe COVID conditions.

Another main reason for successful treatment was enhanced supportive care in this patient. Supplemental oxygen was given to the patient at 4 L/min on the second day of hospitalization. Adequate hydration and nutrition (1500 kcal/day) with nasogastric tube insertion were also administrated to the patient. Finally, patient was put on a prone ventilation position, which was recommended by other experts.^10^ This position would reduce the lung compression, improve oxygenation and improve the lung perfusion.

Active late‐stage prostate cancer patient populations with pre‐existing various high risks are required a thoughtful treatment approach to the COVID infection. To the best of our knowledge, this is the first reported successful case of a patient presented with infection of COVID‐19 delta variant and severe concomitant complications.

## FUNDING INFORMATION

None.

## CONFLICT OF INTEREST

The authors declare that there is no conflict of interest that could be perceived as prejudicing the impartiality of the research reported.
